# Tuberculum sellae meningioma with possible tacrolimus neurotoxicity manifesting as manic-like psychosis after kidney transplantation

**DOI:** 10.1186/s12991-019-0242-6

**Published:** 2019-09-05

**Authors:** Eun Hyun Seo, Seung-Gon Kim, Yong Soo Cho, Hyung-Jun Yoon

**Affiliations:** 1 Premedical Science, Chosun University College of Medicine, Gwangju, Republic of Korea; 2 Department of Psychiatry, Chosun University College of Medicine, 309 Pilmun-daero, Dong-gu, Gwangju, 61452 Republic of Korea; 3 Department of Radiology, Chosun University College of Medicine, Gwangju, Republic of Korea

**Keywords:** Kidney transplantation, Immunosuppressant, Neurotoxicity, Meningioma

## Abstract

**Background:**

Although kidney transplantation is the best treatment option for chronic kidney disease, the accompanying immunosuppressive treatment can induce severe neurotoxicity presenting, on rare occasions, as psychosis. However, a brain tumor synchronous with immunosuppressant neurotoxicity has never been reported in a kidney transplant recipient. Herein, we report the first case of possible tacrolimus neurotoxicity with a meningioma manifesting as manic-like psychosis after kidney transplantation.

**Case presentation:**

A 63-year-old male presenting with acute psychotic mania was admitted to a psychiatric ward approximately 2 years after kidney transplantation. On brain magnetic resonance imaging, a tuberculum sellae meningioma was found, and hyperintense white matter lesions with possible tacrolimus-induced neurotoxicity were seen on fluid-attenuated inversion recovery images. Interestingly, the patient showed no visual field defects, and his blood tacrolimus concentration was within therapeutic ranges. After 3 weeks of adjunctive treatment with blonanserin, most of the symptoms had abated.

**Conclusions:**

The present case highlights the fact that neuroimaging studies are necessary to investigate underlying causes, as well as immunosuppressant neurotoxicity, which should all be considered when atypical psychiatric symptoms develop after organ transplantation. Further, this case suggests that the additional use of atypical antipsychotics while maintaining immunosuppressants may be effective for manic-like psychotic symptoms secondary to possible immunosuppressant neurotoxicity synchronous with a meningioma.

## Background

Kidney transplantation is the treatment of choice for chronic kidney disease as it provides patients with satisfactory quality of life [1]. However, life-long immunosuppressive treatment can induce neurotoxicity, often accompanied with neurological symptoms such as headache and tremor [2]. Rarely, severe neurotoxic side effects presenting as psychosis have been described [3]. Brain tumors, including meningiomas, can cause several types of psychiatric symptoms. Although two cases of meningioma have been reported after bone marrow transplantation [4], secondary brain tumor in solid organ transplant recipients is rare. Herein, we report the first case of possible immunosuppressant neurotoxicity with a meningioma manifesting as manic-like psychosis after kidney transplantation.

## Case presentation

A 63-year-old male with no personal or family history of psychiatric illness was admitted to the inpatient psychiatric ward at a teaching hospital in September 2018 because of sudden-onset manic-like symptoms including irritable mood, increased talkativeness, decreased need for sleep, and hyperactivity that began 2 weeks previously. Concurrently, he was convinced of the existence of a plot by his family members to murder him. He had received a kidney transplant for kidney disease due to diabetes in July 2016. Upon admission, a Mini-Mental State Examination (MMSE) was performed and his score (24/30) indicated impairments in orientation to time and concentration, but no abnormalities were found upon physical and neurological examination. Other than anti-diabetic medications, including gliclazide (60 mg/day) and linagliptin (5 mg/day), the patient was also being treated with the following immunosuppressants: tacrolimus (2 mg/day), methylprednisolone (4 mg/day), and sirolimus (2 mg/day). His tacrolimus levels (6.1 µg/mL) were within the therapeutic range (5–10 µg/mL). The consulted nephrologist recommended continued use of immunosuppressive drugs. On the first day of admission, blonanserin (8 mg/day), an atypical antipsychotic drug, was administered to alleviate psychotic symptoms.

**Fig. 1:**
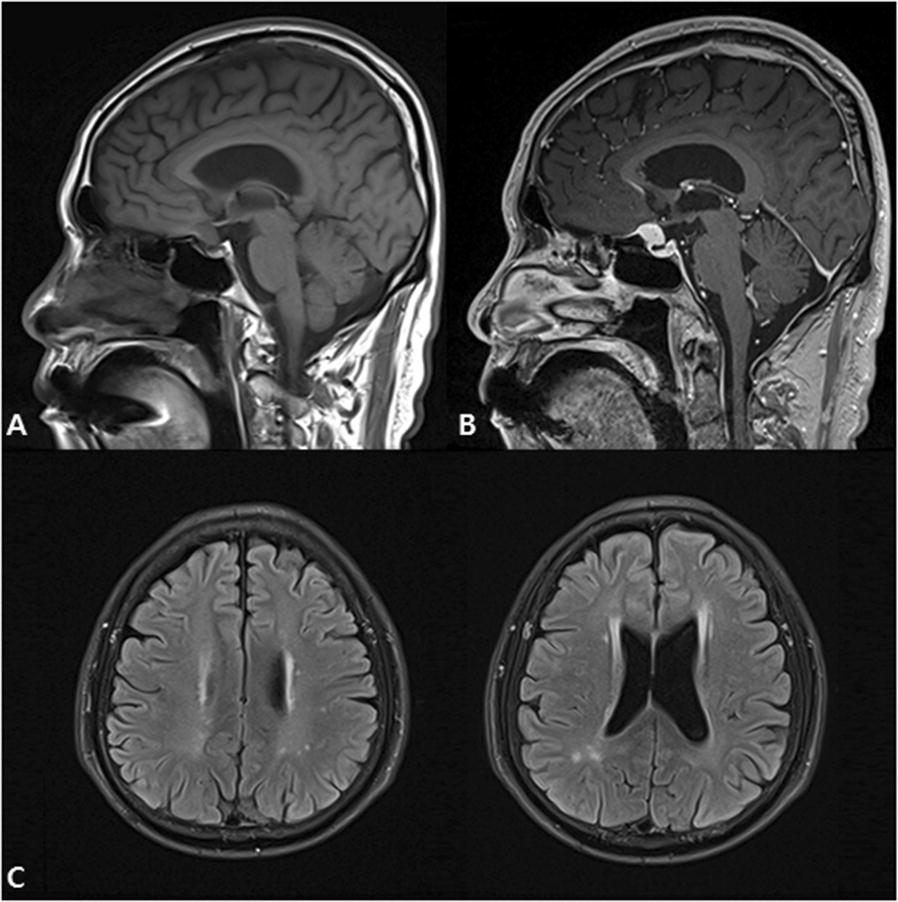
Sagittal T1-weighted (**A**) pre-contrast and (**B**) post-contrast MRI showing a tuberculum sellae meningioma measuring 15 × 8 × 13 mm. Axial FLAIR MRI (**C**) showed multifocal high signal intensities in both the deep and subcortical white matters

On the second day, all laboratory results including a drug screen were normal, except increased serum glucose (220 mg/dL) and hemoglobin A1c (9.7%) levels. However, a cerebral magnetic resonance imaging (MRI) study revealed a tuberculum sellae mass measuring 15 × 8 × 13 mm. The lesion was typical of a meningioma displaying homogenous enhancement with gadolinium and a dural tail (Fig. 1A, B). Hyperintense white matter lesions with possible tacrolimus neurotoxicity were also seen on fluid-attenuated inversion recovery (FLAIR) images (Fig. 1C). Since the patient did not show any visual field defects, neurosurgical consultation recommended regular follow-up without surgery. On the tenth day of admission, manic symptoms and impaired cognitive functions significantly improved (MMSE score: 29/30), whereas persecutory delusions remained unchanged. Accordingly, the patient’s blonanserin dosage was increased to 16 mg/day. Seventeen days after admission, delusions disappeared completely, which was supported by his statement of gaining insight into illness. After 3 weeks of hospitalization, most of the symptoms resolved. He was discharged and kept on the triple immunosuppressive regimen without any change in their doses and a reduced dose of blonanserin (8 mg/day).

## Discussion and conclusions

We present a case of a meningioma synchronous with possible tacrolimus neurotoxicity after kidney transplantation. A previous study reported that kidney transplantation was particularly associated with an increase in cancers with viral etiology, suggesting that immune suppression caused the increased risk [5]. Exposure to radiation is the main risk factor for meningiomas [6], whereas its incidence and risk factors are unknown in immunosuppressed organ transplant recipients. Visual impairments are the main symptoms of tuberculum sellae meningiomas due to compression of the optic chiasm [7]. Interestingly, no visual field defects were found in this case. In a previous study, strong correlations between psychiatric symptom characteristics and meningioma location were found [8]. Frontal convexity menigiomas were mainly accompanied with depression, whereas suprasellar and temporal convexity lesions were accompanied with organic delusional disorder. However, diagnosing brain tumors based on psychiatric symptoms without any neurologic sign can be daunting. A previous retrospective study reported psychiatric symptoms as the initial manifestation of meningiomas presented in 21% of cases occurring in the fifth decade of life [9]. Therefore, a high level of suspicion of brain tumors, particularly of meningiomas, is needed when patients present with atypical psychiatric features. In this case, because our patient did not undergo tumor removal surgery, elucidating the tumor’s causal effect on clinical symptoms is difficult. Despite this, our case emphasizes the necessity of neuroimaging to investigate other underlying causes in cases where immunosuppressant neurotoxicity is highly likely.

Tacrolimus, a calcineurin inhibitor, is one of the mainstays of immunosuppressive protocols in organ transplantation [10]. Calcineurin is downstream of the N-methyl-D-aspartate receptor system and plays a role in regulating dopaminergic neurotransmission [11, 12]. Calcineurin hypoactivity may contribute to schizophrenia development [13]. Based on a consensus report [14], therapeutic ranges of tacrolimus in kidney transplant recipients should be 5–10 µg/mL in maintenance therapy. Although the emergence of neurotoxic symptoms has often been associated with elevated trough tacrolimus concentrations, psychotic symptoms can occur even within therapeutic levels [3]. Therefore, diagnosing neurotoxicity based on tacrolimus concentrations can be misleading. The factors or duration associated with tacrolimus-induced psychosis are also unclear. Appignani et al. [15] reported that predominant neuroimaging findings of tacrolimus toxicity were hyperintense white matter lesions on T2-weighted MRI, suggesting edema. FLAIR is the most sensitive sequence for identifying edema in tacrolimus-induced posterior reversible encephalopathy syndrome [16]. However, normal neuroimaging findings have also been reported in tacrolimus neurotoxicity [3, 15]. Even though it is difficult to verify the direct causal relationship between possible tacrolimus neurotoxicity and prominent psychotic mania-like symptoms, the present case indicates that careful monitoring of psychiatric symptoms should be considered in the management of transplant recipients under immunosuppressive treatment.

Corticosteroids can induce psychiatric adverse events including a variety of affective, psychotic, cognitive, and behavioral symptoms [2]. Patients taking more than 40 mg/day of prednisone or its equivalent are susceptible to serious psychiatric disorders such as steroid psychosis [17]. Other than corticosteroid dosage, reliable risk factors for psychiatric adverse effects have not been identified [18]. Although the patient in this report did not take high-dose corticosteroids, chronic use of low-dose methylprednisolone may also contribute to the development of manic-like psychosis.

When severe psychotic symptoms requiring admission develop under immunosuppressive treatment, discontinuation or dose reduction of immunosuppressants should be considered. In the case of tacrolimus-induced neurotoxicity, switching tacrolimus to another agent such as cyclosporine is an alternative [19]. Moreover, sirolimus did not cause major neurotoxicity when used in heart transplant recipients [20], but switching from tacrolimus can lead to allograft rejection [10]. Although there are no specific guidelines for managing tacrolimus-induced psychosis, a previous case reported successful treatment of psychotic symptoms with an atypical antipsychotic drug without changing the tacrolimus dose [3]. Our case also suggests this to effectively treat manic-like psychosis secondary to possible tacrolimus neurotoxicity with a meningioma. Nevertheless, periodic attempts to reduce or discontinue these agents are necessary to prevent potential adverse events.

This report has several limitations. First, psychotic mania possibly occurred independent of tacrolimus neurotoxicity or meningioma. Because reliable biological markers for bipolar disorder have not been established, additional investigations for differential diagnosis could not be performed. However, some features of the case implied organic causes rather than bipolar disorder. The patient showed the first manic-like episode in his 60s, which is in contrast with the mean onset age of 30 years for bipolar disorder. Compared with over half of patients with bipolar disorder having at least one family member with a mood disorder, our patient did not have any family history of psychiatric illness. Moreover, cognitive deficits such as temporal disorientation suggest an atypical feature of bipolar disorder, which underlines the need to consider another medical condition as a differential diagnosis. Second, although there were no medical abnormalities that could explain the patient’s psychiatric symptoms, it cannot be ruled out that undiagnosed dementia or delirium may have caused cognitive impairments of the patient. Third, given that the pathology of the tumor was not confirmed, it may not have been a meningioma. Fourth, as the patient had not undergone brain MRI before admission, the onset of the tumor is unclear. Further, since a follow-up MRI scan was not carried out, we could not identify the change of hyperintense white matter lesions when the psychiatric symptoms resolved. Finally, considering that silent brain infarcts and white matter lesions are often seen on MRI in neurologically asymptomatic elderly patients [21], lesions observed in our case may have been caused by cerebral small vessel disease. In addition, these white matter lesions and lacunar infarctions could be the potential underlying causes of manic-like psychosis.

In summary, we illustrate the first case of a tuberculum sellae meningioma synchronous with possible tacrolimus neurotoxicity after kidney transplantation, which manifested as manic-like psychosis. Our report demonstrates that neuroimaging studies for other organic causes, as well as immunosuppressant neurotoxicity, are important diagnostic tools when atypical psychiatric symptoms develop after organ transplantation. Further, this case suggests that adding an atypical antipsychotic drug to an immunosuppressive regimen may effectively treat manic-like psychosis secondary to possible tacrolimus neurotoxicity synchronous with a meningioma.

## Data Availability

Not applicable.

## Abbreviations

MMSE: Mini-Mental State Examination
MRI: magnetic resonance imaging
FLAIR: fluid-attenuated inversion recovery
